# Supplementary material for: Specific Neurodynamic Exercises on Pain and Disability in Old Women with Chronic Mechanical Neck Pain: A Randomized Controlled Trial
Source: Healthcare (Basel). 2023 Dec 21;12(1):20. doi: 10.3390/healthcare12010020 (PMC10779372; doi:10.3390/healthcare12010020)
Supplement: Supplementary file 1 [file healthcare-12-00020-s001.zip › healthcare-2781883-supplementary.pdf]

TIDieR

Treatment of chronic cervical pain through neurodynamic exercises.

Why:

Mobilization of upper limb nerves can have effects on pain, disability, pressure pain points, range of motion, upper limb strength, kinesophobia, and catastrophizing.

What (material):

1. They shall consist of neurodynamic median, ulnar and radial nerve gliding exercises and exercises of the main entrapment points described in thoracic outlet syndrome. The movement exercises will be performed 15 repetitions 3 times with 30 second breaks. Stretching exercises will be performed 1-2 times maintaining 1 minute.

What (procedures):

Table B1. Explanation of the exercises performed in the neurodynamics group.

Opening of the cervical foramen conjunctiva

Craniocervical and cervical flexion: perform correct craniocervical flexion and maintain it while performing full neck flexion.

Cervical lateroflexion: perform neck bends without shoulder rise.

Stretching of the scalene muscles

Stretching focused on stretching the medial and anterior fascicles because the plexus passes between them. Performed with slight head extension and fixation of the shoulder stump downwards, the head is brought into a contralateral tilt and homolateral rotation.

Opening of the costoclavicular space

The aim is to descend the rib and ascend the clavicle. To do this, while performing a deep and continuous exhalation (costal descent), an elevation of the stump and an external glenohumeral rotation are performed.

Stretching of the pectoralis minor:

In a standing position, place the right forearm and hand resting on the frame of a pillar, the elbow should be higher than the shoulder and the forearm with an abduction of about 130 degrees (slightly higher than the shoulder stump), with external rotation and extension component until the tension is felt.

Serratus anterior activation:

Both hands are placed on a wall at shoulder height and retropulsions and antepulsions of the shoulder are performed.

Neurodynamic sliding of the median nerve

The parameters were: descent, external rotation and abduction of the shoulder, straight elbow, supination of the forearm, dorsal flexion and extension and opening of the fingers. Once the parameters have been set, the glide is performed by tilting the head towards the arm while maintaining elbow extension. In the other direction, the neck is tilted towards the other side and the elbow is flexed while maintaining the rest of the parameters.

Neurodynamic sliding of the radial nerve:

The parameters were: descent, internal rotation and abduction of the shoulder, elbow in extension, pronation of the forearm, palmar flexion and flexion of the fingers. Once the parameters have been established, the slide is performed by tilting the head towards the arm while maintaining elbow extension. In the other direction, the neck is tilted towards the other side and the elbow is flexed while maintaining the rest of the parameters.

Neurodynamics of ulnar nerve gliding:

The parameters were: descent, external rotation and abduction of the shoulder, flexion of the elbow, supination of the forearm, dorsal flexion of the wrist and closed fist. Once the parameters were established, the slide was performed by tilting the head towards the arm while increasing elbow flexion. In the other direction, the neck is tilted to the other side and the elbow is extended while maintaining the rest of the parameters.

**Who provided:**	The intervention was carried out by physiotherapists with more than 1 year of experience dealing with older people providing exercise. It was done without material, only giving the orders to the participants while performing the exercises.
**How (mode of delivery; individual or group):**	Both the assessment and the intervention were carried out in person by both the participants and the physiotherapist. Two assessment visits were carried out where all the study variables were collected. And each participant completed 12 exercise sessions spread over four weeks with a duration of 50 minutes.
**Where:**	The assessments were carried out in consultation 2 of the Policlinica FisioEnUSAL of the Faculty of Nursing and Physiotherapy of the University of Salamanca. It has optimal conditions to be able to carry out the evaluations. And the exercise sessions were held in the senior centers belonging to the Salamanca city council.
**When and hoy much:**	wo assessment visits were carried out where all the study variables were collected. And each participant completed 12 exercise sessions spread over four weeks with a duration of 50 minutes. The movement exercises will be performed 15 repetitions 3 times with 30 second breaks. Stretching exercises will be performed 1-2 times maintaining 1 minute.
**adaptation:**	As explained in the article, the movements were personalized based on the neural tension and pain that each participant showed. The movement and exercise were the same for everyone.No increase in load was required throughout the intervention.
**Modification:**	N/s
**How well (planned):**	All participants completed the 12 exercise sessions except for the 3 people who did not complete the program. Modifications were made to all of them in positions so that the neural tension was always present. Adherence to the program was complete and sessions were completed as planned.
**How well (real):**	Adherence to the program was complete by all participants who completed the program. Only 3 people (5%) did not finish the program. The intervention went as planned.
